# A Review of Norms and Normative Multiagent Systems

**DOI:** 10.1155/2014/684587

**Published:** 2014-07-09

**Authors:** Moamin A. Mahmoud, Mohd Sharifuddin Ahmad, Mohd Zaliman Mohd Yusoff, Aida Mustapha

**Affiliations:** ^1^College of Information Technology, Universiti Tenaga Nasional, Jalan IKRAM-UNITEN, 43000 Kajang, Selangor, Malaysia; ^2^Department of Software Engineering, Faculty of Computer Science and Information Technology, Universiti Tun Hussein Onn Malaysia, Parit Raja, 86400 Batu Pahat, Johor, Malaysia

## Abstract

Norms and normative multiagent systems have become the subjects of interest for many researchers. Such interest is caused by the need for agents to exploit the norms in enhancing their performance in a community. The term norm is used to characterize the behaviours of community members. The concept of normative multiagent systems is used to facilitate collaboration and coordination among social groups of agents. Many researches have been conducted on norms that investigate the fundamental concepts, definitions, classification, and types of norms and normative multiagent systems including normative architectures and normative processes. However, very few researches have been found to comprehensively study and analyze the literature in advancing the current state of norms and normative multiagent systems. Consequently, this paper attempts to present the current state of research on norms and normative multiagent systems and propose a norm's life cycle model based on the review of the literature. Subsequently, this paper highlights the significant areas for future work.

## 1. Introduction

The term social norms is used to define the behaviors of society members. Social norms as defined by Cialdini and Trost [1] are “rules and standards that are understood by members of a group and that guide and/or constrain social behavior without the force of laws.” According to Melnyk [2], these rules and standards entail the expected value of others that can be identified by observing their behaviors. Basically, social norms are informal rules and standards which are socially shared and comparative stable guides of society members' behaviors. However, the informal and nonobligatory character implies the presence of social reinforcements, such as agreement or disagreement, and discriminate social norms from laws [2].

Norms usually direct the option of behaviors in human communities. Conformity to norms reduces social frictions and facilitates coordination [3]. Norms manage a variety of phenomena, involving “property rights, contracts, bargains, forms of communication, and concepts of justice,” and regulate a uniform behavior within a social group but often differ substantially among groups [4]. Over time, norms changes could happen, due to objective circumstances or changes in subjective perceptions and expectations [4].

The objectives of this paper are (i) to review and discover the current state of norms architecture and the normative processes, (ii) to propose a norm's life cycle model based on the current state of norms research, and (iii) to propose potential future work in norms and normative multiagent research. Our contribution in this paper is threefold. Firstly, it proposes norms taxonomy. Secondly, it defines a new type of regulative norms and thirdly, it proposes a norm's life cycle model.

The next section reviews the literature on social norms, specifically, in the definition of norms in social science and multiagent systems. It first presents the two main norms classification as proposed by the literature, which are conventional and essential norms. Subsequently, it discusses several norms characteristics such as concepts, definitions, types, and finally the norms' life cycle. This is followed by a comprehensive review of normative multiagent systems in Section 3.

Section 4 presents a comprehensive review of the empirical studies in normative systems such as norm creation, enforcement, spreading, emergence, detection, and assimilation and their simulation mechanisms. Having presented the different empirical studies on normative systems, Section 5 reviews the models in the literature on norm's life cycle. From the review, we discuss the limitation of each model, based on which we develop our own norm's life cycle model which extends and augments the findings of existing models.  Section 6 presents suggested future work and Section 7 concludes the paper.

## 2. Social Norms

### 2.1. The Concept of Norms

Norms (in this paper, norms and social norms have the same meaning) are informal rules that are socially enforced. However, a norm represents the expected behavior towards a specific situation [5]. The concepts of norms are used to determine the behaviors of agents within a community and are commonly accepted as efficient means to normalize their behaviors [6]. Norms represent desirable behaviors for a population of a natural or artificial community and they are generally understood as rules indicating actions that are expected to be pursued that are either obligatory, prohibitive, or permissive based on a specific set of facts. According to Hollander and Wu [7], norms have been used to indicate constraints on behavior [8], to create solutions to a macrolevel problem [9], and to serve as obligatory [10], regulatory, or control devices for decentralized systems [11].

Anderson and Taylor [12] categorize norms into three main kinds:

- folkways: they are not substantially important norms and only mildly enforced in a society, that is, right manners, suitable dress, and proper eating behavior;
- mores: they are the important norms of a society; mores violation evokes strict punishment (against the law most of the time), for example, incest and cannibalism;
- laws: the type of norms which are designed, maintained, and enforced by the political authority of a society, for example, speeding, cheating on income tax, and murder.

The literature has presented ample definitions of norms [13]. Hexmoor et al. [14] suggested that “a norm has different definitions in different areas of study such as social science, game theory, psychology, and legal theory.” The Webster's Dictionary (http://www.webster.com/) defines a norm as [15]

- an authoritative standard;
- a principle of right action binding upon the members of a group and serving to guide, control, or regulate proper and acceptable behavior;
- generally as
  - a set standard of development or achievement normally derived from the average or median achievement of a large group;
  - a pattern or trait taken to be typical in the behavior of a social group;
  - an extensively practiced procedure or custom.

These definitions represent the term of norm in different disciplines of normative research such as sociology, psychology, philosophy, deontic logic, legal theory, decision theory, and game theory [15]. Based on Verhagen [15] and Hollander and Wu [7], we set out the following definitions.

- In sociology and social theories, norms are rules or constraints of behavior that are socially enforced and considered valid by the majority of a social group [4, 13, 16–18]. Tuomela [19] distinguished different kinds of norms which are rules (r-norms), social norms (s-norms), moral norms (m-norms), and prudential norms (p-norms). Rules (r-norms) depend on agreement of authority (e.g., taxes have to be paid by all), social norms depend on mere mutual belief (e.g., people should not spit), and moral norms appeal to one's conscience (e.g., one should not steal or cheat). Prudential norms are based on rationality (e.g., one has to maximize one's expected utility) [20].
- In deontic logic, norms are represented as obligations or permissions that an individual has to a larger social system [21]. Obligation can be in an opposite form, in which situation it is indicated as prohibition [7].
- In legal theory, norms are any rules of behavior imposed by an authorized body and enforced via applying sanctions [10].
- In decision theory, game theory, and any other theory that depends on rational actors handling norms in a similar way, a behavior that has been adopted by the majority of a social group is considered successful [16].

The website, http://changingminds.org/ (2008), defines norms as behavioral rules that are used for suitable and unsuitable values, beliefs, and attitudes in a social group. Savarimuthu et al. [22] define norms as expected behaviors by the members of a specific society.

### 2.2. Fundamental Norms

The fundamental norms are driven by injunctive norms, which refer to people's beliefs about what have to be done [23] and descriptive norms, which refer to beliefs about what is really done by the majority in one's social group [24]. This could be exemplified by a formal meeting, in which a majority of the attendees are silent and attentive (descriptive norms), so much so that others act in a similar manner fearing the incurrence of social sanctions such as frowning or giving silent gestures if they do not comply (injunctive norms) [24].

Melnyk [2] argued that descriptive norms (what a majority of population does) influence behavior directly, while injunctive norms (what the population approve) could activate attitudes. They support their argument with results that show that

- descriptive norms possess a stronger influence on behavior than injunctive norms;
- descriptive norms possess a weaker influence on attitudes than injunctive norms.

### 2.3. Norms Classification

In the literature of normative multiagent systems, several terms have been used to present the concept of norms, which are conventions, social norm, and social law [25]. Coleman [26] defined two main categories of norms: conventions and essential norms. Correspondingly, Villatoro [25] grounded the difference between conventions and essential norms.

*Conventions*. Conventions are natural norms that emerge without any enforcement [25]. Conventions solve coordination problems when there is no conflict between the individual and the collective interests; for example, everyone conforms to desired behavior [25]. Young [27] defined conventions as “a pattern of behavior that is customary, expected, and self-enforcing. Everyone conforms, everyone expects others to conform, and everyone wants to conform given that everyone else conforms.” Conventions fix one norm amongst a set of norms which is always efficient as long as each one in the community employs the same norm, that is, greetings, driving side of the road [25]. 

*Essential Norms*. Essential norms solve or ease collective action problems when there is a conflict between an individual and the collective interests [25, 28]. For example, “the norm not to pollute urban streets is essential in that it requires individuals to transport their trash, rather than dispose of it on the spot, an act that benefits everyone” [29].

The literature of normative multiagent systems suggests three kinds of norms [30]. The first kind is regulative norms, which specify the ideal and varying degrees of subideal behavior of a system by means of obligations, prohibitions, and permissions [30]. The second kind is constitutive norms, which normalize the creation of institutional norms, in addition to the revision of the normative system itself [31]. The third kind is the procedural norms, which are instrumental norms addressed to agents acting on roles in the normative system intending to perform the social order, particularly in terms of substantive norms [32].

*Regulative Norms*. Regulative norms are intended for regulating activities by imposing obligation or prohibition in performing an action [33]. As Peczenik [34] commented, a regulative norm qualifies an action or a state of affairs as prescribed, permitted, or prohibited. Because regulative norm qualifies an action, it can be treated as a norm of conduct, for example, the responsibility to lodge a police report without a reasonable delay upon finding lost or stolen things. A norm of conduct can prescribe punishment or a sanction for a person who violates a norm. One can thus make a distinction between a sanctioned and sanctioning norm. Peczenik [34] also described moral norms that serve goal norms, for example, the guarantee that “everybody ought to have a decent standard of living.” In other accounts, regulative norms produce regulation of an earlier problematic situation by setting rules for actors' behavior, which represent obligation and prohibitions [35], for example, the rule that a person “should drive on the right lane” [33]. 

*Constitutive Norms*. Constitutive norms are affirmed to produce new goal norms or states of affairs, for example, the rules of a game like chess [33]. Boella and van der Torre [36] observed numerous features of constitutive norms; one of them is an intermediate concept exemplified by a statement, for example, “this is a presiding official in a wedding ceremony” or “this bit of paper counts as a five-euro bill” [36]. Two other features are organizational and structural norms, which refer to how roles define power and responsibilities and how hierarchies structure groups and individuals [30]. Norms are introduced by both the agents who play legislative roles as well as ordinary agents who create new obligations, prohibitions, and permissions concerning specific agents [31]. Boella and van der Torre [36] differentiated regulative norms and constitutive norms with an example; for instance, if the regulative norm states that vehicles are forbidden in the park, then the constitutive norm is that “bicycles are also counted as vehicles in the park.” 

*Procedural Norms*. Procedural norms are categorized as objective and subjective. Objective procedural norms represent the rules that express how decisions are really made in a normative system, while subjective procedural norms represent the instrument for individuals working in a system, for instance, back-office procedures [37].

### 2.4. Norm Life Cycle

The literature on social norms reveals that there is no unified view on the mechanism of norms creation and spreading in a society or social group [38–40]. However, according to Coleman [41], “norms are macrolevel constructs based on purposive actions at the microlevel but coming into an existence through a micro-to-macro transition. Once in existence, they lead, under certain conditions, to actions of individuals (i.e., sanctions or threat of sanctions) which affect the utilities and thus the actions of the individuals to whom the sanctions have been or might be applied.”

## 3. Normative Multiagent Systems (Normas)

Boella et al. [42] claimed that researchers of moral and legal philosophy have studied traditional normative systems [43]. Such systems have been integrated with multiagent systems when numerous models on multiagent systems have been investigated that include norms in agent architectures [44–46].

According to Savarimuthu [40], norms in multiagent systems research can be traced back two decades ago [8, 47–50]. Normative multiagent systems are combination of two established fields which are normative systems and multiagent systems [42]. They are represented by sociological theories in multiagent systems applications and by the relation of agent theory and the social sciences such as sociology, philosophy, economics, and legal science [36]. The concepts of normative multiagent systems are used to facilitate cooperation and coordination among social group [8, 51, 52].

Jones and Carmo [53] defined a normative multiagent system as “sets of agents (human or software) whose interactions can be regarded as norm-governed, whereby the norms prescribe how the agents should and should not ideally behave.” Boella and van der Torre [32] defined normative multiagent system as “a multi-agent system organized by means of mechanisms to represent, communicate, distribute, detect, create, modify, and enforce norms, and to deliberate about norms and detect norm violation and fulfillment.”

Researches in NorMas have proceeded widely and ardently within this decade [5]. Some suggested concepts by researchers involved attribution on mental attitudes to normative systems [54]; defining the role of a defender agent to achieve the task of detecting violations and sanctioning them on behalf of a normative system [31]; obligation and permission [55]; formalizing the triaspolitica using the standard BDICTL logic for agent verification [56]; regulative and constitutive norms [31]; substantive and procedural norms [32]; norm implementation [57]; and a verification framework for normative multi-agent systems [58]. Other researchers focus on norms construction in institution and formalizing relationship between norm and agent's behavior [59] and providing a specification of the desired overall normative system behavior [60].

### 3.1. State of the Art in Normative Systems

In the architecture of normative multiagent systems, the literature offers ample research information on normative systems. We present here several frameworks and their description. 

*BOID Normative Architecture*. Broersen et al. [44] proposed the belief, obligation, intention, and desire (BOID) architecture, which is the BDI architecture with an obligation component, O. It has feedback loops to consider all effects of actions before committing to them and mechanisms to resolve the output conflicts of its components. BOID determines logical criteria to retract the attitudes of agent with the changing environment and to resolve conflicts by stating different general policies according to the considered agent type. Agent types conform to such ways by which conflicts are identified and settled. A realistic agent thus corresponds to a conflict-resolution type in which beliefs override all other factors, while other agent types, such as simple-minded, selfish, or social ones, adopt different orders of overruling. 

*Programming BOID-Plan Agents*. Dastani and van der Torre [61] introduce an abstract and operational semantics of an agent programming language which is used to implement cognitive agents. They modeled the mental attitudes which are represented by rules based on the BOID architecture. By monitoring the environment, the agent can generate goal sets from desires, obligations, and intentions, select goals, generate plans, and execute them. To program the agent's deliberation process, these actions can be combined in the deliberation language in different ways. At the level of abstraction, goal generation and planning are both characterized as conflict resolution procedures. 

*BIO Normative Architecture*. Governatori and Rotolo [62] proposed a BIO architecture, which considers three components (beliefs, intentions, and obligations). BIO follows the BOID (belief, obligation, intention, and desire) architecture to describe agents and agent types in defeasible logic. But there are some peculiarities that make it different from other frameworks such as BOID. Particularly, the system develops a positive account of those modalities that match to mental states and obligations. Rules are thus meant to devise appropriate logical conditions for introducing modalities. The development of social agents focuses on the components of obligation and intention. The agents' compliance could be tested by directly focusing on plan design and execution [62]. 

*Normative KGP Agents*. Sadri et al. [45] presented a framework that demonstrates how normative concepts, such as obligation and prohibition, can be used by an agent while it reasons, reacts, plans, and communicates in the context of an artificial society. The framework builds upon an existing framework called the KGP (knowledge, goals, and plans) model of agency which they implemented in the prototype agent platform PROSOCS. They develop agents that can reason about norms that are expected to govern their own behavior while pursuing their own goals. 

*OP-RND Normative Framework*. Ahmad et al. [46] developed a normative agent framework called the obligation-prohibition-recommended-neutrality-disliked (OP-RND) framework to regulate rules and norms effectively. Their agents perform tasks from a set of precompiled tasks based on their beliefs of the reward and penalty associated with the selected tasks. They define obligation, O, as a command imposed by some agent in authority. In such environment, an agent is obligated to perform an action and gets rewarded for doing it or penalized for leaving it. Prohibition, P, is defined as a command, in which the agent has to avoid an action and hence gets rewarded for leaving it or penalized for doing it. They consider obligation and prohibition (OP) as rules imposed by the authority in a normative environment due to absolute consequences (reward or penalty) upon conformation or violation of some action [46].

### 3.2. Norms Representation in Agent-Based Systems

In normative systems, norms used in agent-based systems must be presented in a manner that allows them to be processed by software agents [7]. According to Savarimuthu [40], researchers have represented norms in both explicit and implicit data structures. Hollander and Wu [7] refer to four major representation schemes that have been used in recent research which are deontic logic, rule-based systems, binary strings, and game theory [7].

- Deontic logic is developed from modal logic that is an expanded version of classical formal logic which deals with the “necessary” and “possible”. While, deontic logic deals with obligations, prohibitions, and permissions [63, 64].
- Rule-based systems are sets of condition/action pairs coded with an inference engine. It is usually used by systems that take benefit of offline design, where the norms are represented implicitly into the agent's decision making system [8, 20, 48, 65–68].
- Binary strings are orders of ones and zeros digits, where the digit one represents the occurrence and digit zero represents the absence of a norm. This format is often used in research on population to test the transmission and emergence of norms [69–73].
- In game theory, every agent is able to make a simple choice that yields a corresponding payoff and in every round it tries to maximize their payoff by taking an action based on what they expect their adversary to take [7].

### 3.3. Open Normative Multiagent Communities

According to Savarimuthu [40], an open normative multiagent community means that norms are not explicitly given to a visitor agent. In other words, the agent is not conferred with the community's norms in offline mode. Instead, the agent must be able to identify the norms by using some detection algorithm.

While this work concurs with the authors in this definition, it proposes the idea that an open community also refers to the agent's free and unrestricted movement from one community to another to achieve its goal. In such situation, when the agent visits a new community and it does not have any knowledge about the community's norms, it should be equipped with algorithms for detecting the norms.

### 3.4. Norms Enforcement in Normative Systems

To motivate an agent to comply with the domain's norms, the norms are enforced by sanctions. According to Hollander and Wu [7], enforcement is used during and after the spreading process to create a motivation for agents to adopt a new set of norms and ensure that agents keep obeying the acquired norms [18]. Enforcement can be directed externally, internally, or motivationally [4].

Normally, a third-party enforcement agent is given the ability and authority to implement the sanctions [74]. In addition, it avoids agent from norm violation by applying sanctions [75]. But noncompliant norms could also trigger emotions of shame or guilt in an agent even when a third party enforcement is absent [76]. This fact is especially efficient in large-scale communities, where it may be difficult to monitor compliance with equilibrium behavior that entails sanctions by a third party [4].

### 3.5. Discussion

In this section we discuss three issues that we consider as deficiencies in the norms literature.

**Figure 1:**
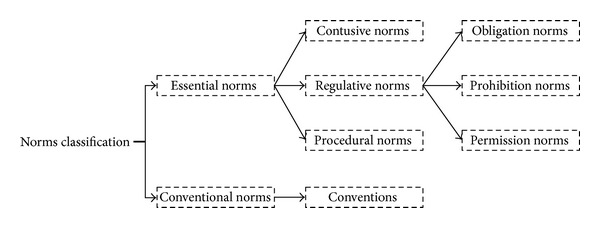
Norms classification architecture.

(i) The first issue is that the literature classifies norms into conventional and essential norms and reveals that there are three types of norms which are constitutive, regulative, and procedural norms. In our perspective, the three types of norms are located under essential norm because these types represent the definition of essential norms which is solving collective action problems in case of conflict between an individual and the collective interests by applying reward or penalty.  Figure 1 shows the norms classification based on the literature. However, in this work we focus more on regulative norms which directly affect agent behaviors [30]. Consequently, we only include regulative norms types in Figure 1 and in the rest of this discussion.

With regard to the definition of regulative norms, the literature defines regulative norms to constitute the obligation, prohibition, and permission norms, as follows.

- Obligation norm is the norm that may cause reward or penalty; that is, if an agent exercises the norm, it avoids the penalty, but if it does not do so, it is penalized [46]. From the definition, we can infer the case that when an agent acts on an event, it avoids a penalty and when the agent does not act on an event, it gets a penalty.
- Prohibition norm is the norm that may also cause penalty but in a negated sense of obligation norms; that is, if the agent does not exercise the norm, it avoids the penalty, but if it does it, it is penalized [46]. Intuitively, from the definition, we can also infer the case that when an agent acts on an event, it gets a penalty and when the agent does not act on event, it avoids a penalty.
- Permission norm is part of obligation norms but it exempts agents from some of their obligatory behavior under specific circumstances [77].

From the previous discussion, we conceive another case of regulative norm that has not been deliberated in the literature. This is the situation when an agent gets rewarded for exercising a norm but is not penalized otherwise. Consequently, we propose a new type of regulative norms, which we called the recommendation norms. We adapt the term “recommendation” from the OP-RND framework by Ahmad et al. [46]. In their work, they divide agent performance into three mutually exclusive periods: recommended (R), neutrality (N), and disliked (D). Recommended period in this framework represents a period when an agent is rewarded if it completes a task within this period but is not penalized otherwise. In this work, we adapt the definition of recommended norms to represent any actions or behaviors of agents that are judged by the community as noble or altruistic, hence merit for rewards. This is the type of norm that rewards an agent if it is exercised by the agent but is not penalized otherwise.

A case for the recommendation type norm is that when an agent acts on an event, it gets a reward and when the agent does not act on an event, it gets no reward or penalty.

To clarify the idea of recommendation norms, we offer the following example. Consider a scenario in a crowded elevator and someone, S, wants to choose a floor but is unable to reach the buttons, while another person, X, is standing just besides the buttons. There are two situations:

1. X offers to help S and presses for him/her the desired floor button and S rewards X by thanking him/her;
2. X does not offer to help S but S does not penalize X.

**Figure 2:**
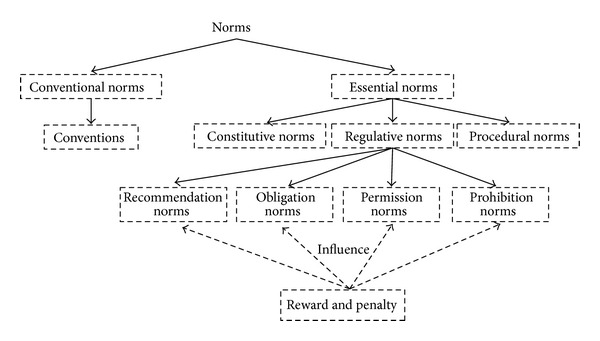
Modified norms classification architecture.

**Figure 3:**
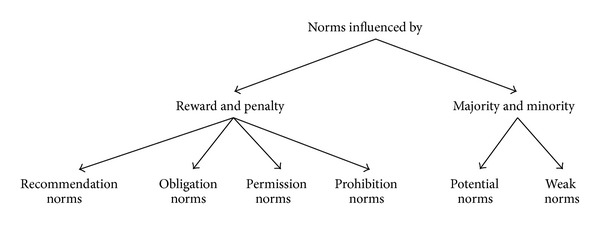
The structure of norms' influence.

Figure 2 shows the modified norms classification architecture.

(ii) The second issue is the regulative norm types (i.e., recommendation, obligation, prohibition, and permission). We argue that these types are influenced by reward and penalty only and they are not applicable to domains that are not applying reward and penalty. We claim that there is another way to describe those norms' domains based on population adoption. Since the norms are enacted by the majority of a population [7], we consider that there are two types of norms which are potential norms and weak norms. The potential norms are adopted by the majority of the population, while the weak norms are adopted by the minority. The norms can be influenced by reward and penalty and by the majority and minority of population adoption.  Figure 3 shows the structure of the norms' influence.

(iii) The third issue is the empirical work on norm life cycle of which there are several [7, 38, 40]. We argue that all of these works have missed to include norms assimilation as a mechanism of norms enforcement and emergence. We shall discuss this issue in Section 5 in detail and present our perspective by modifying the existing work on norm's life cycle.

## 4. Empirical Research and Mechanisms on Norms

The next few sections present a comprehensive review of the empirical studies in normative systems and their simulation mechanisms and discuss the limitations of these studies. We then review the models in the literature on norm's life cycle.

### 4.1. Norms Creation

The process of presenting a new norm in a normative system is called norm creation [7]. According to Posner and Rasmusen [78], norm creation requires spreading of the norm and developing sanctions for its violation. In the real world, norms are created from three methods which are, natural emergence from social interaction, decree by a powerful agent, and agents negotiation within a group [20, 21, 38, 79].

**Figure 4:**
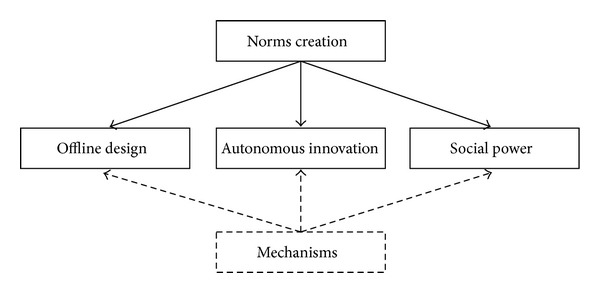
Norms creation mechanisms.

Savarimuthu [40] claimed that there are three approaches to create a norm in artificial intelligence agents: when the norms are specified by a designer [48], when the norms are specified by a leader [15, 49], and finally when the norms are considered good for society by a norm entrepreneur [80]. However, Hollander and Wu [7] defined two approaches in general which are offline design and autonomous innovation. Savarimuthu [40] defined another approach, which is social power.  Figure 4 shows the mechanisms of norms creation.

#### 4.1.1. Offline Design

In offline design, designers encode norms directly in agents, which enact the norms. Any new norms required by the system in future are updated by the designers. This approach could be practically implemented in simple systems, but, in complex reasoning systems, offline design could fail in capturing the details required for realistic performance [7]. Another limitation of this mechanism has been specified by Savarimuthu [40] who objected to the notion that offline design assumes that all agents adopt the norms in a society which might not be realistic especially in open communities, when different norms are competed to present as the society's norm.

An example on offline design is by Shoham and Tennenholtz [47] who tested traffic-associated norms [40]. Other examples are by Walker and Wooldridge [51]; Conte and Castelfranchi [48]; and Hales [68] who created experiments based on the offline design approach. Conte and Castelfranchi [48] simulated agents finding foods in a grid in which agents are engaged in some basic rules for movements and food collections. In this work, they assumed that the agents are made up of strategic agents or normative agents. In this simulation, the strength of an agent is increased when it consumes food, while it is reduced when it moves from one cell to another in the grid. From their simulation that compares utilitarian and normative strategies, they discovered that norms decrease the violence level and increase the average strength of an agent.

#### 4.1.2. Norms Autonomous Innovation

In this approach, agents create new norms without any external interference. For this to happen, the challenge of ideation must be addressed. Ideation is how an idea of behavior becomes a norm in the first place and filtering which ideas are accepted and rejected [7, 17].

Current researches on norms creation based on innovation (ideation and filtering) have focused on machine learning and game theory [20, 81]. In game theory, ideation is almost reduced to offline design and filtering is based on the choice of the most successful behavior. Such situation holds true for machine learning in which ideation is conceived via search and filtering is also based on the selection of successful behavior. In more advanced situation, Andrighetto et al. [82] suggested an alternative approach based on cognitive architecture for research on norms creation via simulation which allows more exploration on norm innovation [7, 82, 83].

#### 4.1.3. Social Power Mechanism (Leadership and Punishment)

Social power can also be an important notion in establishing norms [84–86]. López [87] noticed that an agent is able to express its social powers via its ability to change the beliefs, motivations, and goals of other agents. The sources of power can either be leadership mechanism (encourages and motivates followers to adopt a particular norm) or punishment mechanism (enforces others to follow a particular norm) [40]. Another approach by Boman [49], which is based on centralized advisor, proposed that an agent is consulted with a normative advisor before performing any action.

### 4.2. Norm Emergence

The term “emergence” is used to describe norm creation and establishment on a microscale [7]. The literature provided several definitions of norm emergence. Finnemore and Sikkink [38] defined it as “persuasion by norm entrepreneurs which try to convince a critical mass of states (norm leaders) to embrace new norms.” Another definition by Hollander and Wu [7] described that the norm is considered emerged when it has been adopted by an adequate number of agents in a society. Savarimuthu [40] suggested that when the norms have reached some significant threshold in the degree of norm spreading, it indicates that the norms are followed by a substantial proportion of agents in the society.

Ample researches have been conducted in the area of norm emergence [3, 11, 40, 88–92]. Hollander and Wu [7] categorized the literature on norms emergence within normative multiagent systems into three main areas. The first area uses the game theory to describe the dynamics of norm emergence. The second area investigates the relationship between sanctions and norm emergence and the third area attempts to realize the effect of transmission on norm emergence.

Sen and Airiau [3] suggested a model of social norms emergence by learning from interaction experiences. In their model, each agent interacts repeatedly with other agents in the society and every interaction is considered as a stage game. The learning process in this model (which leads to norm emergence) is that any agent in the game can identify the policy of the game from repeated interactions with multiple agents. They term this learning mode as social learning from repeated interactions against players. They investigated the effect of size of population, number of actions, different learning strategies, nonlearning agents, and the norms' evolution speed and stability in multiple relatively isolated populations. Their results show that when the interaction probability is at least 0.3, only one norm pervades the whole population and when the interaction probability is 0.2, less divergent norms emerge.

Brooks et al. [90] proposed a theoretical approach to study the dynamics of agents population playing a coordination game to specify the whole norms to which the society can converge. They developed a prediction system of linear repetition relations that shows (i) how frequently every norm will be reached and (ii) the average time of convergence. The study aimed to examine the norms emergence process and predict the possible final norm that emerged. They validated their prediction model for both constant and proportional bias update schemes by using the empirical results from a large number of simulations. They proved that a population using one of these two update rules (constant and proportional bias update) almost definitely converged on one of a small set of norms.

Hollander and Wu [92] described a model of group norm emergence. In this model, they presented a simulation model of multiagents built on top of the model of norms emergence. Based on the simulation, they presented screening experiments on the simulation that is aimed at observing the significant factors that contribute to the emergence of group norms and consensus formation. The experimental results show that (i) the model can attain consensus as well as two additional states of information equilibrium, (ii) both network structure and agent behavior play an important role in the formation of consensus, and (iii) the formation of consensus is sensitive to the simulation parameter settings and certain values can prevent its formation entirely.

### 4.3. Norm Enforcement

In agent communities, norms are used to regulate agents' behaviors but agents may decide not to comply with the norms if this benefits them. Consequently, norms enforcement is designed to offset these benefits and thus the motives for not complying with the norms [75]. Social enforcements are often used to enforce an agent to adopt the behavior of other agents [7]. Enforcement can be performed externally, internally, or motivationally [4]. However, norms enforcement as defined by Savarimuthu [40] is the process of discouraging the violators of the norms via some form of negative sanction such as punishing and encouraging the followers of the norms via some form of positive sanction such as reward. These processes help to sustain norms in a society.

**Figure 5:**
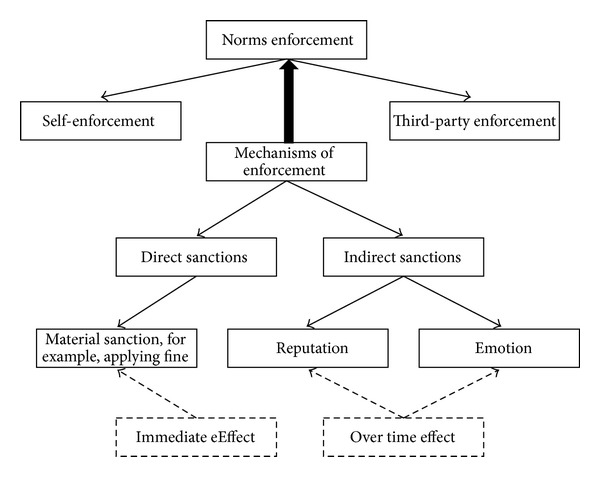
Types and mechanisms of norms enforcement.

To perform the enforcement, it requires a process that is able to detect the activity of the norms and their probable violations and handle this violation [93]. According to de Pinninck et al. [75], norms enforcement can be achieved through a controller via stopping forbidden actions or agent controller via applying reward and penalty on agents.  Figure 5 shows the types and mechanisms of norm enforcement.

#### 4.3.1. Self-Enforcement

Self-enforcement is also called as internally directed enforcement [7]. It occurs when an agent punishes itself for violating a norm, which could happen when an agent has internalized the norm and is influenced by some emotion. According to von Scheve et al. [94] and Staller and Petta [95], emotion is one of the critical and important factors that drive self-enforcement [7].

In self-enforcement, the violator performs its own penalty and this is often because its actions are not coordinated with the actions of other agents. In other words, there is no third party involved in its actions to apply punishment [78]. However, in case of self-enforcement, an agent is a victim of a norm violation and is involved in the punishment without prior information [75].

#### 4.3.2. Third-Party Enforcement

The literature referred to this type as externally directed enforcement [7]. A third-party enforcement agent has the ability and authority to implement the sanctions (reward or penalty) [74]. By applying sanctions, it prevents an agent from directly involving in a norm violation [75].

Third-party enforcement occurs when an agent observes another agent violating a norm [11, 71, 73] or during norm spreading when an agent does not adopt the norms of others. As a result, the observing agent or an associated authority applies sanction on noncompliant agent [7]. In agent communities, externally imposed sanctions are often used to constrain a deviant or undesirable behavior and reduce the overall deviance or undesirable behaviors in the population [69].

#### 4.3.3. Mechanisms of Enforcement

In normative systems, designing an enforcement mechanism is considered a very important issue in agent communities where agents might violate the expected behavior. These enforcement mechanisms can be exploited to enforce agents towards compliance with the norms and these mechanisms are usually monitored to discover norm violations and trigger sanctions [96].

To enforce an agent to adopt or quit a certain norm, it needs to provide mechanisms to motivate the agent to follow or avoid the norm. The mechanism can be enforced internally based on shame or guilt, motivationally based on reputation, or externally based on sanction such as reward or penalty [7].

Several kinds of sanctions have been defined in the literature, that is, sanctions based on emotion and reputation, or can take some forms of relationship damage such as a loss of trust or friendship [7, 13, 40, 75]. However, according to Cardoso and Oliveira [98], there are two basic kinds of sanctions, which are direct material sanction that has an immediate effect (e.g., by applying fines) and indirect social sanction, which may have an effect that extends over time, that is, changing an agent's reputation. 

*Direct Sanction*. The coercive action against others to enforce them to follow socially is known in the literature as a sanction [7]. A sanction can be any form of punishment for noncompliance with the associated norms or some form of reward for compliant agents [7]. The sanction is applied by either a neighboring agent who observes a deviant agent or by some forms of authority structure that detects and sanctions the violators [7]. The sanction is often associated with a cost such as breakdown in relationship and loss or stop working in some of utility value [7, 13]. There are two common strategies used when applying direct sanctions, which are deterrence to discourage any future violations by punishing the violator and retribution that intends to compensate the victim of the violation [98].

Axelrod [99] used an enforcement mechanism called metanorms that shows punishing agents who did not comply with the norms and achieves norms equilibrium in the society. The simulation of the metanorms mechanism also shows that there is an increasing norm stability in every run of the experiment. However, he discovered that when the punishment associated cost is low for the punishers, the norm can be sustained [40].

Vázquez-Salceda et al. [100] studied the problem of developing a mechanism for enforcing norms and they proposed a sanction mechanism which provides services to support police agents to enforce proper behavior. The police agents are not able to observe the internal information and process of the other agents, but they enforce the norms based on the detection of public actions of violators. They apply some forms of sanction (e.g., black lists, clock triggers, and action alarms) to simplify norm enforcement on multiagent communities [100]. 

*Indirect Sanction*. Indirect sanction can be influenced via agent's reputation or agent's emotion, whose effects extend through time. Two explicit sanctions are identified in a reputation mechanism: a positive opinion towards an agent when it complies with society norms or a negative opinion towards the agent when it violates the society's norms [40, 101]. The concept of reputation has been widely used in several works [101–106] but the term in the different approaches holds different semantics. In other words, there is no unified view about the meaning/semantics in the different approaches [101].

According to Grizard et al. [101], some researchers assume that each agent has only one reputation globally handled by the system [107], while others consider that two agents can possess a different view about the reputation of an agent [103, 105, 106]. Others think that reputation is related to the given context [102] of the sources exploited to develop their target nature [108]. However, Casare and Sichman [109] attempted to present a unified view of all the mentioned aspects based on functional ontology of reputation [101].

The work by Grizard et al. [101] suggested that there is no globally maintained reputation value; therefore, two agents can maintain different reputation values for the same target. Thus, the values of reputations are maintained by other agents and are external to the sanctioned agent.

The value of an agent's reputation decreases if the agent violates the norm (negative sanction) or increases if the agent conforms to the norms (positive sanction). They consider that the sanctions based on changes in reputation are motivated to respect the norms for the violating agents because one of the costs for an agent in low reputation might be the refusal of other agents to interact with it (social exclusion). They formalize the sanction in terms of reputation as “sanction (applier; sanctioned; weight)” where applier is the agent who applies the sanction, sanctioned is the agent that is sanctioned, and weight is the value of the sanction. The weight parameter is used to affect the reputation value of an agent according to a specific mechanism.

Emotions have also been used in normative systems research to sustain social norms over time [95, 97] and von Scheve et al. [94]. For example, people feel embarrassed if they violate social norms (e.g., wearing jeans in a formal dinner) [46, 95, 110].

Staller and Petta [95] presented an emotion mechanism based on the work of Conte and Castelfranchi [48]. In their work, they showed that the computational study of social norms can benefit by modeling emotions among agents in artificial communities. Consequently, they suggested the TABASCO architecture for the development of appraisal-based agents. Bazzan et al. [111] constructed a framework for simulating agents with emotions, by employing a scenario that regards social norms for agents. Furthermore, they used the OCC model for the computational model of cognitive and behavioral features of emotions.

**Figure 6:**
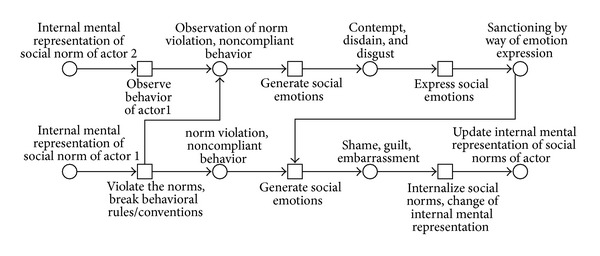
Internalization, adapted from Fix et al. [97].

Fix et al. [97] present a Petri net-based model of sanctioning noncomplying behavior by methods of social emotions. In their scenario (as shown in Figure 6), there is a violator (actor 1) and an observer/punisher (actor 2). Actor 2 observes the behavior of actor 1 and attempts to discover a norm violation. As soon as violation has been discovered, the emotions of disdain, scorn, or revulsion are elicited (transition “generates social emotions”) and their expression (transition “expresses social emotions”) comprises the punishment of a violator agent which lead to (place “sanctioning by way of emotion expression”) the negative emotions in the violator (transition “generates social emotions”) and induce states of shame, guilt, or embarrassment.

### 4.4. Norms Detection

Norms detection is the process of updating an agent's norms based on discovering a society's potential norms through some detection mechanisms which rely on observing or interacting with other agents to infer the potential norms. According to Hollander and Wu [7]; Boella et al. [112]; Conte and Dignum [113], when researchers attempt to build a normative multiagent system, norms detection is one of the main challenges faced by the designer. The literature provides other terms of norm detection such as norms recognition, norms adaptation [7], and norm identification [40].

Hollander and Wu [7] defined norm recognition as the agent's ability to observe or interact with a group of agents and discover the right norms of the agents in that group. In case of humans, they are often able to accomplish that via conversation [114]. However, norm recognition is also concerned about the ability to detect deviant agents within a group [7]. Hollander and Wu [7] have also defined norms adaptation as a process of adapting new norms in which system's norms change over time. According to Savarimuthu [40], norm identification mechanism can be exploited when the norms have not been explicitly created in the society. An agent can identify the norms from its environment through interactions with other agents.

**Figure 7:**
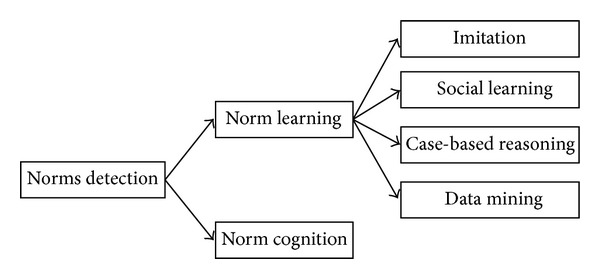
Norms detection approaches and mechanisms.

Norm detection is inspired by the process of norm learning [7, 40, 115] and norm cognition [40]. Several studies have been made by researchers on norm learning based on mechanisms of imitation [70, 87, 116]; social learning [3, 117–120] case-based reasoning [121]; and data mining [122–124]. Others have worked on norm cognition [82, 123, 124].  Figure 7 depicts the norms detection mechanisms derived from the literature.

#### 4.4.1. Norm Learning

Norm learning is the ability of learning from others and it is an active technique to complement and support the learning of individuals. In particular, norm learning presents the basis for culture where norms are spreading within society and pass down from one generation to another [125]. As we have mentioned earlier, there are four mechanisms of norm learning suggested by the literature.

*Imitation Mechanism*. Epstein [70] proposed an imitation model based on adopting the behavior of a majority of population. The phenomenon of imitation has been described as “when in Rome, do as the Romans do.” This model is based on the local environment state and the amount of thinking of agent regarding its behavior.

Lòpez [87] justified the needs of learning mechanism in normative system because agents make decisions based not only on their motivations and own goals, but also on their observation of the normative behavior of other agents. The author proposed three strategies to influence agents to comply with a norm of related actions of other agents. The strategies are simple imitation, reasoned imitation, and reciprocation.

Andrighetto et al. [116] presented a comparative study between two models of learning that are validated by simulations, which are learning-based imitation and learning-based recognition. The simulation study attempted to compare the normative agents' behaviors provided with (i) a norm recognition module, which they called norm recognizers (NRs) and (ii) a social conformity population model, called social conformers (SCs), whose behavior is specified by imitation rule. 

*Social Learning*. Social learning in agent society means that each agent learns from repeated interactions with other agents in a society [3]. The individual's behavior is largely influenced by the interaction with others, through social learning [119]. Sen and Airiau [3] proposed a social learning theory, in which every agent in the community learns simultaneously from repeated interactions with randomly selected neighbors. The key to success of this method depends on how an agent learns from other agents within the social network.

Bosse et al. [119] presented a dynamic agent-based approach to simulate and formally analyze the process of social learning of agents' behaviors. The general mechanism is based on behavior changes by influence of peers. The approach involves the influence of three types of agents groups which are peers, parents, and school.

*Case-Based Reasoning*. Campos et al. [121] used case-based reasoning (CBR) as a learning technique to decide how to adapt domain-level norms that depend on current system status. CBR learning is based on heuristics that aligns the amount of serving/receiving capacity, and this heuristic is used by the CBR to suggest a solution when no similar cases are found.

*Data Mining*. Few studies in norms detection emerged from data mining applications. Data mining entails scouring through data records in databases to identify significant patterns that are useful for a decision-making process [126]. Among the data mining tasks such as classification or clustering, association rule mining is one particular task that extracts desirable information structures like correlations, frequent patterns, associations and casualness between sets of items in transaction databases or other stores of data [126, 127]. Association rule mining is widely used in many different fields like telecommunication networks, marketing, risk management, inventory control, and others [126]. The association rule mining algorithm discovers association rules from a given database such that the rule satisfies a predefined value of support and confidence. The aim of using support and confidence thresholds is to ignore those rules that are not desirable, because the database is huge and users care about those frequently occurring patterns only [128].

Symeonidis and Mitkas [122] presented an agent-oriented algorithm that deals with agent actions, which is called K-profile. K-profile is mainly used to predict an agent's behaviors by exploiting data mining techniques to extract the knowledge from historical data and express the actions of agents within the multiagent systems.

Savarimuthu et al. [123, 124] develop two algorithms; the one to identify obligation norms is called Obligation Norm Identification (ONI) [124] and the other to identify the prohibition norm is called Candidate Norm Inference (CNI) [123]. These two algorithms are designed based on data mining, specifically on the association rule mining approach.

#### 4.4.2. Norm Cognition

In recent research on norm detection, a new approach has been suggested by Andrighetto et al. [82], which is norm cognition or cognitive approach. According to Savarimuthu [40], the cognitive approach shows potential because agents based on this approach have the normative expectation notion. Specifically, cognitive approach focuses on what happens inside an agent's mind to detect norms when they join new communities and deliberate about norms. Agents based on this approach can propose a new norm that relies on their past experience [40].

Andrighetto et al. [82] proposed a norm innovation theory in coping with specific types of complex entities such as a social system called the EMIL architecture. Two-way dynamics are categorized by the theories which are emergent processes consisting of emergence from interaction among individual agents and emergent effects: emergence of entities (norms) at the aggregate level into the agents' minds.

Savarimuthu et al. [123, 124] emphasized the importance of the cognitive approach and presented a cognitive model, in which agents are located in a domain where other agents entering the domain may not be aware of the protocol associated with domain's norms. An agent located in the domain is able to observe other agents' actions and is able to extract the society's norms from these actions based on the ability of recognizing negative and positive signals (e.g., reward and penalty) events by using a filtering algorithm. The agent, after identifying the normative protocol of the society, updates its personal belief base by adding or removing norms.

### 4.5. Norms Spreading

**Figure 8:**
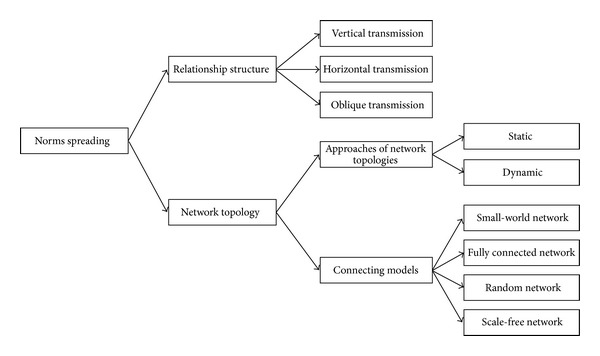
Norms spreading relationships and network topologies.

The process of distributing norms in a society or social group is called norms spreading [40]. Hollander and Wu [7] defined norms spreading as “the ability for norms to spread is a consequence of the system's underlying network topology in conjunction with active and passive transmission mechanisms.” However, the spreading mission is to transmit a norm from an agent to another [7]. There are several ways to spread norms and to cover that we discuss the spreading process via two main subjects which are relationship structure and network topology.  Figure 8 shows the details.

#### 4.5.1. Relationship Structure

According to Savarimuthu [40] and Hollander and Wu [7], there are three ways by which a social norm can spread between society members which are vertical transmission, horizontal transmission, and oblique transmission [17, 129].

*Vertical Transmission*. The case that describes the kind of vertical transmission is the norm transmission from parents to offspring [7, 40]. The spreading process based on vertical transmission ensures that the offspring adopt some or all of their parent's norms; in other words, it generates offspring that inherit parents' behavior and thus ensures the transmission of norms from one generation to another [7, 40, 130]. According to Savarimuthu [40], one famous research in this subject is by Axelrod [99] and other researchers who have tested the vertical model for norm spreading [131, 132].

*Horizontal Transmission*. Another kind of spreading is horizontal transmission [7], which occurs when the norms are transmitted between peer interactions in the same generation [133, 134]. The advantages of these laterally spreading norms through a population are to enable agents to adopt new norms from their unrelated neighbors and to increase the variety of an agent's behaviors during its own lifetime [7].

*Oblique Transmission*. The last kind of norms spreading based on relationship is oblique transmission and an example of this kind is the transmission of the norms from a leader of a society or social group to the followers [7, 40]. Oblique transmission occurs when norms are broadcasted from an authority body to a set of subordinates and this process can spread the norms both vertically and horizontally. This approach is used by centralized multiagent systems and normative systems [7, 80].

Savarimuthu [40] suggested that vertical and oblique transmissions can be considered as leadership mechanisms that encourage the followers to acquire norms and horizontal transmission can be considered as peer-to-peer mechanism where agents learn from daily interactions with other peers.

#### 4.5.2. Network Topology

Researches in network topology of normative multiagent systems are mainly related to the effects of agent topology on the norms spreading or emergence [7]. In norms spreading, the social network between members in real world is very important because people are not related to each other randomly, but they are connected via the social groups such as work groups, ethnic groups, and hobby groups [40].

In social systems [135], the network topology is essential to study the different social phenomena based on networks properties and characteristics. For example, Macy and Willer [136] noticed the importance of network density and they discovered that increasing network density increases the difficulty of coordination. Borgatti and Foster [137] described the homophily phenomenon which indicates people's tendency to interact with other similar people based on individual characteristics such as shared beliefs [135].

**Figure 9:**
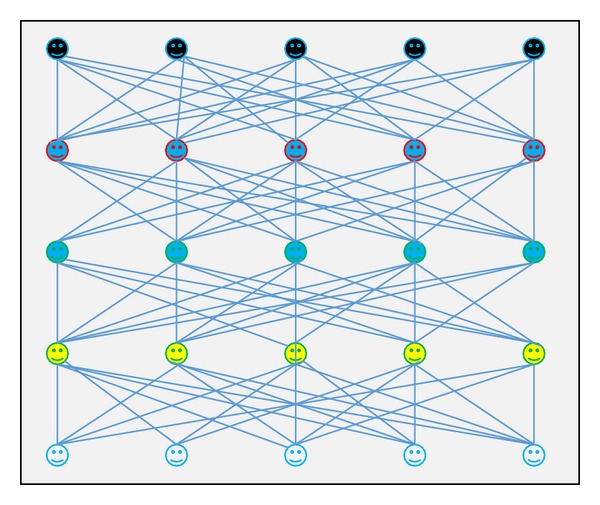
Network topology, adapted from Macal and North [138].

There are two approaches of network topologies which are static and dynamic (Figure 9). In case of static topologies, the topology is fixed; in other words, the links are prespecified. For dynamic topologies, the fundamental network can change and the links are determined endogenously based on the mechanisms involved in the model [40, 138]. There are several ways or structures for agents to be connected with each other. The connecting models are a small-world network, fully-connected or complete network, a random network, and a scale-free network [40, 91, 135, 139].

*Static and Dynamic Network Topology*. According to Fan and Ammar [140], when the required communication is fixed through time, the ideal choice of network topology is static, whereas when the required communication is changing through time, the ideal choice of network topology is dynamic. However, the problem of static topological design has been widely studied for native networks. The dynamic topology design did not get the same attention because through small time scales, the hard-wired native networks are normally not reconfigurable [140].

In case of static network topology, agents interact between them depending on their location in a circular lattice [40]. Several researches have been conducted on the static network topology [3, 10, 40, 132, 141]. The work by Kittock [141] was the first experimental study on the role of network topology in emergence of convention [40]. Kittock [141] noticed that the choice of global structure has a high impact on the system evolution and this is based on the network topology and the convention emergence varies. Specifically, he supposed that the network diameter is directly related to the convergence rate [40]. Shoham and Tennenholtz [88] developed an algorithm called Highest Cumulative Reward (HCR) algorithm that helps an agent to learn about choosing the best interaction strategy within a social network.

Nakamaru and Levin [72] conducted several experiments on the evolution of two norms by using four different types of network topologies. The two norms are the background of an agent against the opinions that the agent holds. The background is a norm that is shared by the population but agents in the population can hold different opinions about the background norm. They note that (i) when people of similar background meet, some of their opinions might change (ii) and when two agents have similar opinions and different backgrounds, they could change their background [40].

Few researches have been conducted on the dynamic network topology [40, 142]. Griffiths and Luck [142] studied the emergence of norms between agents in a network topology, in which agents rewire their links with immediate neighbors by swapping their worst neighbors with the best neighbors.

Savarimuthu [40] conducted experiments on the role model mechanism for norm emergence that works on top of dynamically evolving networks. In their simulation, the set-up of experimental architecture for norm emergence comprises the social network topology and the role model mechanism and the networks are constructed based on the mobile agent model of Gonzaléz et al. [143]. To perturb the network, the links are changed (adding and removing links).

In another work, Fenner et al. [144] presented a social network in stochastic model. The model represents the network dynamic nature as it evolves through time. Agents may join the network; existing actors may inactivate themselves and reactivate at a later period. Actors manage new relations based on a preferential attachment rule that weights different agents according to their degree [139].

**Figure 10:**
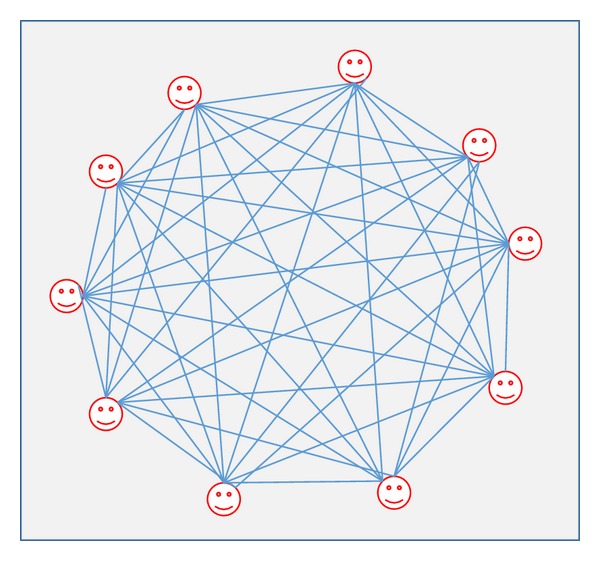
Fully-connected network, adapted from Ross et al. [135].

*Connecting Models*. There are different possible social ways of connection between individuals in a society or social group. As we have mentioned earlier, the most famous ways are a fully connected or complete network, small-world network, a random network, and a scale-free network [40, 91, 135, 139]. In fully connected or complete network of a specific society, every agent is connected to all other agents in the society as shown in Figure 10.

This case might be represented when an organization is rather small and each one communicates regularly and works closely with other agents. In such case, each agent influences all other agents and relies on the rule of local influence, and the agents attempt to reach the same values of belief through time [135]. Savarimuthu [40] claimed that numerous researchers have experimented this type of topology and most of these experiments include interactions with all the society's agents [10, 49].

**Figure 11:**
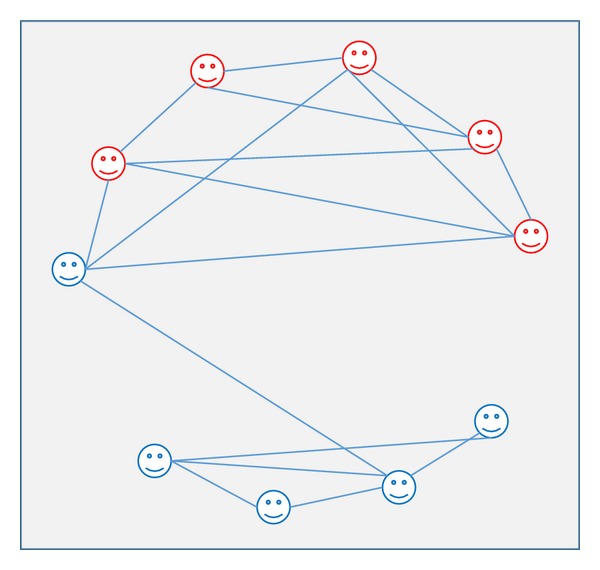
Small-world network, adapted from Ross et al. [135].

The second type of network topology (as shown in Figure 11) is the small-world network [135], which hypothesizes that each agent in the network is connected to all other agents by only a few steps [145]. For example, in sociology, based on observation on individuals' relations, most people have many friends living nearby, but also they have a few friends living far away [145]. A similar example by Ross et al. [135] suggested that, within organizational society, people might have many friends in their department, while they have a few friends in other departments.

**Figure 12:**
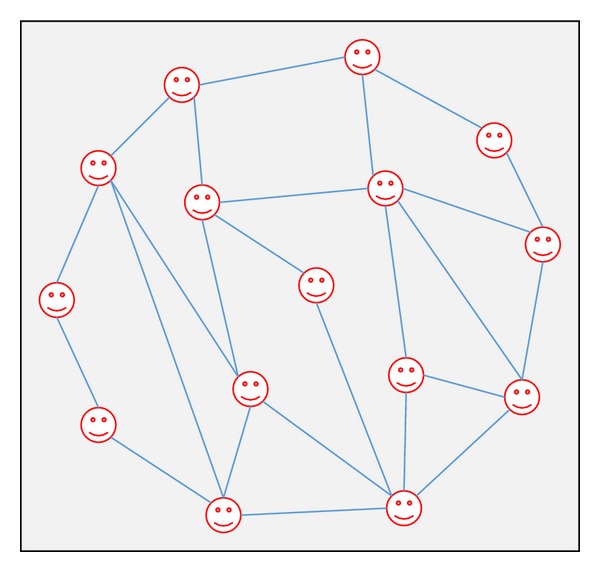
Random network, adapted from Savarimuthu [40].

The other type is a random network (as shown in Figure 12), in which links are established randomly between nodes based on some probability distribution which must be less than 1 because if the probability equals 1, the network becomes fully connected [40]. The probability of nodes connecting in the work by Ross et al. [135] is based on a binomial distribution, which means that the agent who connects with other agents has no greater probability of being connected to the two randomly selected agents within the network. Erdös and Renyi [146] studied the random graphs properties and have presented random networks generating mechanism [40].

**Figure 13:**
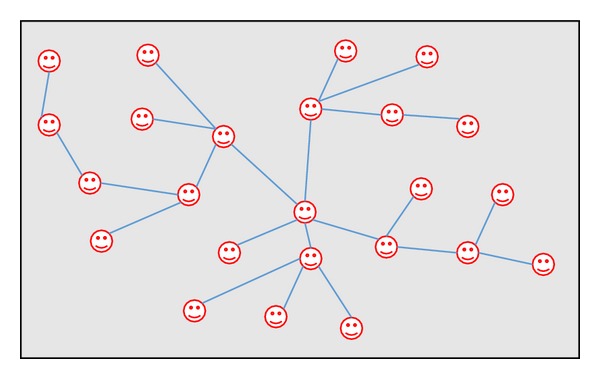
Scale-free network, adapted from Savarimuthu [40].

The last type of network topology is a scale-free network (as shown in Figure 13). Nodes in such network are not connected to each other randomly, whereas a few nodes are well connected and are called hubs and other large number of nodes connected to a few nodes only. It is called scale-free because the ratio of well-connected nodes to the number of nodes in the rest of the network remains constant as the network changes in size [40].

According to Ross et al. [135], in scale-free network, the distribution of nodes is based on law of power which means that there are a few nodes that have a large connection (high power) and relatively many nodes that are sparsely connected (low power). In this topology, the highly connected (high power) nodes play a main role in carrying the other nodes of the network close to each other [135]. Barabási and Albert [147] presented a mechanism for generating a scale-free topology based on their observations of large real-world networks, that is, the Internet, social networks, and protein-protein interaction networks [40, 148].

### 4.6. Norm Internalization

Internalization is the process in which agents integrate information (new norms) into their cognitive structure [7]. Conte et al. [149] defined norm internalization as a mental process that acquires norms as inputs and presents them to the internalizing agent's new goals as outputs. Once the norm has been adopted by agents, social enforcements continue to enforce into internal desires and motivations and ensure that agents continue complying with the norm. Through time, the norms are incorporated into the desires of agents and priority shifts from the original norm possessed by agents to the newly acquired norm (internalization) [7]. In general, internalization can be considered as the measurement unit of compliance towards the performance of new norms [150]. The steps that an agent should perform to internalize a norm are as follows [7].

*(1) Norm Acceptance*. The norms to be internalized must be accepted by an agent. Norm acceptance is the process of conflict resolution where external enforcements on the agent vie against its internal desire. This happens when there is a conflict with existing norm or the associated cost of accepting is too high that it is rejected [151]. However, as long as the norm can be removed, it is possible for the agent to experience new norm conflict with the existing norm.

*(2) Transcription*. When an agent has accepted a norm, it then must go through a transcription process. Transcription is the process of adding new norm to the agent's knowledge base.

*(3) Reinforcement*. After a norm has been accepted and integrated with an agent, it then goes through reinforcement process to ensure that the agent is obeying the norm. Failing in obeying the norm subjects the agent to sanction that forces it to reevaluate its behavior and adopt new norms.

Few researches have discussed norm internalization [15, 149, 152–154] and the subject becomes more popular after the presentation by Andrighetto and Conte [152]. However, most existing studies on normative systems consider a norm as internalized as soon as it has been adopted [7].

Verhagen [15] used a simulation model to measure norms internalization and spreading. The simulation model consists of a group of agents with one of them acting as the leader. The agents roam in a two-dimensional space. The measurement method of norm internalization is by determining the difference between an agent's self-model and its group model, and if there is no difference, then the agent has internalized the norms.

Andrighetto et al. [154] presented and implemented an internalization module that has been integrated into EMIL-A agent architecture [82, 83]. The implemented experiments observed internalizer behavior (internalizer is the agent who has internalized the norm) in communities with different types of agents when a norm is salient and nonsalient. The salient norm means providing information to people about the behavior and beliefs of other individuals. Their results show that a norm is salient EMIL-I-A (EMIL internalizer agent) and goes through all the internalization stages and when the norm is no more salient, it returns to its normative behavior.

### 4.7. Norm Assimilation

Crudely put, norms assimilation is the process of joining and abiding by the rules and norms of a social group. Eguia [155] defined assimilation as the process in which agents embrace new social norms, habits, and customs, which is costly but offers greater opportunities. The problems of norms assimilation are attributed by the ability and capacity of an agent to assimilate in a heterogeneous society, which entails a number of social groups that have different normative protocols (in compliance and violation) and the motivation required for the agent to assimilate with a better-off group [155].

A literature search within the domain of norms and normative systems does not seem to produce a substantial number of research papers that discuss the empirical approach to norm assimilation. The papers only discuss the meaning of the word “assimilation” without building any concrete concept about it [30, 116, 156]. However, the concept of assimilation has been discussed in the domain of social sciences deliberating on the assimilation cost between two social groups concerning the difference in assimilation costs between better-off and worse-off groups or between minority and majority groups [155, 157, 158].

### 4.8. Norm Removal

Norm removal is the ability of removing an obsolete norm and replacing it with a new norm which occurs when there is a conflict between the domain's new norm and an internalized obsolete norm of an agent. The removing process is theoretically important when the system has been updated and becomes more complicated or it is limited in resources [74]. However, there are no any particular researches on the outcome of norm removal except few processes that are often implicit in many systems that implement norm modification [7].

### 4.9. Discussion

From the review and analysis of norm processes and mechanisms, we notice several limitations and gaps in the work of norms and normative systems. There is an obvious gap in research in norm detection. Norm detection is critical in overcoming the problem that occurs from its absence in research, which is the offline design of norms [5, 48, 51, 68]. In particular, the gap is more obvious in the area of norm detection based on the cognitive approach where very few researches have been conducted in this area [40, 82].

The second limitation is the clear gap in norm assimilation. There is no literature found in this area except a few research work from social studies [155, 157, 158]. However, the works by social science researchers are neither developed for the normative multiagent systems nor for the evaluation process of norm's life cycle in agent communities. But the results of these research can be exploited to build an assimilation approach that offer a useful contribution in the domain of normative multiagent systems.

Other limitations are research gaps found in norm internalization and norm removal for which the literature does not provide ample and significant research output.

## 5. The Evolution of Norm's Life Cycle

There are ample researches on normative multiagent systems with considerable amount of structure, similarity, and connectivity to manifest the fundamental organization of norms that enables researchers to create a process-oriented model of norm's life cycle [7]. In this section, we review the literature on evolutionary process of norm's life cycle, derive the deficiencies of each model, and conclude the discussion with a comprehensive norm's life cycle.

### 5.1. Suggested Models of Norm's Life Cycle in the Literature

In the previous sections, we presented the various normative processes and mechanisms that have been used by researchers to build normative systems (e.g., norm creation, norm emergence, etc.). This section reviews and presents the suggested models on the evolution of norm's life cycle as described in the literature. Three main studies have been found in the literature on norm's life cycle. The first work is by Finnemore and Sikkink [38] and the two latest works are by Savarimuthu [40] and Hollander and Wu [7].

Finnemore and Sikkink [38] identified a three-staged process of norm's life-cycle.

- The first stage is norm emergence by persuading other agents to follow the norm.
- The second stage is norm cascade that includes wide norm acceptance specified by imitation, which attempts to socialize others to become followers.
- The third stage is norm internalization when a norm in a society is widely accepted and becomes a routine task for the followers.

The first two stages are divided by a threshold point, at which a critical mass of relevant actors adopt the norm. The characteristic mechanism of norm emergence is conviction by norm entrepreneurs which persuade a critical mass of norm leaders to embrace new norms. Norm cascade is characterized by imitation of the norm leaders and their strong motivation but the norm that cascades through the rest of the population may vary. Norm internalization is characterized by the adoption of a norm by the majority and it is no longer a matter of broad public debate. However, completion of the life cycle is not a certain process because many of norms emergent fail to reach a threshold point [38].

Savarimuthu and Cranefield [20] identified four main phases of norm's life cycle which are norm creation, norm spreading, norm enforcement, and norm emergence. For each specific phase, some simulation mechanisms are assigned. However, Savarimuthu [40] updated the phases and their simulation mechanisms to become five phases as follows:

- norms creation, in which multiagent systems' norms are created by one of the three mechanisms which are offline design, leadership, and entrepreneurship;
- norms identification, which is active when the norms have not been explicitly created and agents need a mechanism to identify the norms based on interactions with other agents, and two mechanisms have been suggested to identify norms which are learning and cognition: learning can be achieved via imitation, machine learning, and data mining;
- norms spreading is concerned with norms distribution within a social group; several mechanisms can help in norms spreading such as leadership and cultural evolution;
- norms enforcement is the process that discouraged violating norms or encouraged the practice of norms by society members via some forms such as sanction, reputation, and emotion;
- norms emergence is a process in which a norm has been adopted by a substantial proportion of a society and is recognized by the majority.

Another work by Hollander and Wu [7, 92] identified several normative processes of norm life cycle which are creation, transmission, recognition, enforcement, acceptance, modification, internalization, emergence, and forgetting. The three main processes are called superprocesses which are enforcement, internalization, and emergence.

In this work, the norm evolution proceeds as follows.

- Norms are primarily created from ideas.
- New norms are then spread via active or passive transmission.
- Agents' neighbors are exposed to the new norms. In this stage, social enforcement is required to ensure that those norms are adopted and internalized.
- Internalization means the new norms are shifted from agents' original preferences to the newly acquired norms.
- This chain of transmission, enforcement, and internalization is known as normative emergence.
- The norm disappears and becomes invalid when existing norms are no longer suitable to the current conditions. They are candidates to be removed and new norms are created via an evolutionary process.

### 5.2. Discussion

Having presented the available research in the literature on evolutionary process of norm's life cycle, this section discusses the gap in each model and concludes the discussion with another novel norm process that fills the gaps in the existing norm's life cycle.

The model by Finnemore and Sikkink [38] begins from norm emergence while other researches on norm's life cycle start with norm creation followed by norm emergence [7, 40]. Another issue is that norm enforcement has not been clearly presented in the proposed model. In addition, other processes such as norms detection and norms assimilation have not been mentioned or defined.

The model by Savarimuthu [40] does not include three processes which are norm internalization, norm assimilation, and norm removal. Norm internalization is a very important process that is required for an agent to embrace a new norm [7, 38]. Norm assimilation completes the process of norm detection or identification that he suggested in his work. It is futile for an agent which has identified a norm but has no mechanism to assimilate with a social group or society [155]. Finally, norm removal is also an important process to start a new cycle of evolution [7].

We concur with the model proposed by Hollander and Wu [7, 92], although their work has not discussed norms assimilation. In the next section, we attempt to develop a model that includes the processes that are missed in the literature such as norms assimilation.

### 5.3. A Proposed Norm's Life Cycle Model

Our proposed model in this section is inspired by the existing models in the literature [7, 38, 40, 92]. In the proposed model, we consider new processes such as norm assimilation, norm detection, and norm adoption. We also offer a new structure that we believe to be closer to reality. However, the main processes of the proposed model are norm creation, norm emergence, norm assimilation, norm internalization, and norm removal. Norm emergence entails several processes, which are called emergence operation. Emergence operation comprises norm enforcement and norm adoption, while norm adoption includes norm detection and norm spreading processes.

**Figure 14:**
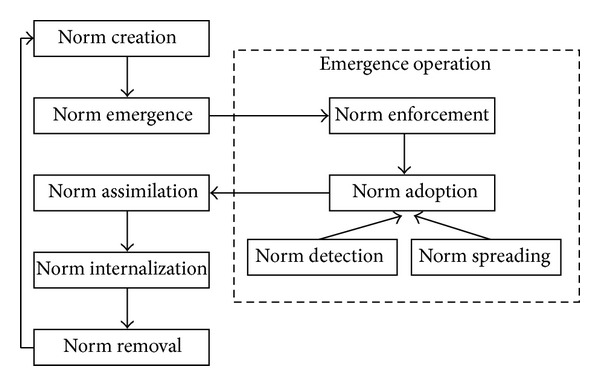
A proposed norm's life cycle model.

As shown in Figure 14, the cycle begins from the norm creation process as the first stage of the life cycle. The second stage is norm emergence that is accomplished through another operation, which we called the emergence operation. The emergence operation starts by enforcing the norms via some enforcement mechanisms. During enforcement, agents adopt this norm through the processes of detection or spreading. They then assimilate the new norm within their social group. Finally, when the agents have assimilated the norm, they proceed with the norm internalization process to establish the norm. The life cycle ends when the norm is no more valid for some reasons and is removed and replaced with a new norm.

**Figure 15:**
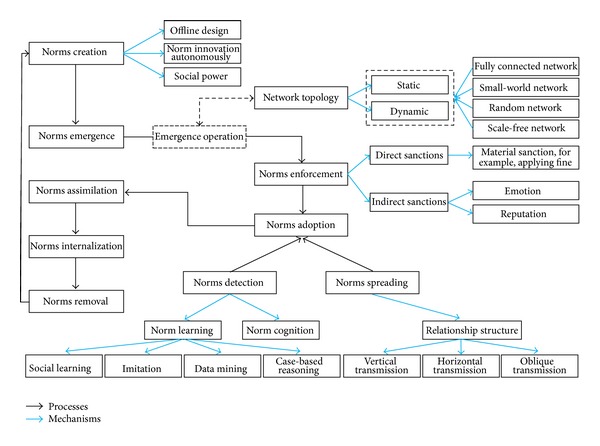
The proposed model with mechanisms.

Consequently, we set out each process and its available mechanisms that have been discussed in Section 4. The structure is categorized according to our proposed model as shown in Figure 15.

- Norm creation: the mechanisms that are associated with this process are offline design, norm autonomous innovation, and social power.
- Norm emergence: it can be accomplished via the emergence operation which entails the following processes and mechanisms:
  - Norm enforcement: the mechanisms that are associated with this process are direct sanction and indirect sanction. Direct sanction is represented by a sanction (reward or penalty). Indirect sanction is represented by reputation or emotion.
  - Norm adoption: it can be achieved through two processes, norm spreading and norm detection as follows:
    - norm spreading has three mechanisms based on relationship which are vertical transmission (e.g., from parents to offspring), horizontal transmission (e.g., peers interactions), and oblique transmission (e.g., from leader to followers),
    - norm detection which is inspired by norm learning and norm cognitive approaches. Four mechanisms of norm learning have been defined by researchers, which are imitation, social learning, case-based reasoning, and data mining.
  - The implementation of norm emergence is based on network topology.
  - Network topology: it is divided into static and dynamic topologies and entails four models of connection which are fully connected network, small-world network, random network, and scale-free network.
- Norm assimilation: having new norms detected by agents, they then calculate the assimilation cost and accordingly decide whether to assimilate or decline.
- Norm internalization: in this process, agents integrate the new assimilated norms into their cognitive structure.
- Norm removal: norms which are considered obsolete by a social group or society are removed from society members' cognitive structure.

## 6. Suggested Future Work

The scope for research in norms and normative multiagent systems paves the way for many exciting new discoveries that could be integrated in physical agents or robots. We outline here some interesting areas that could be investigated in our future work.

*Norm's Context Awareness*. From our review of the literature, we discover that agents are not aware of the context of the enacted norms. Consequently, it would be interesting to look into semantic agents that can deal with ontology-based contexts. When agents could understand the meaning of the norms, they would have greater reasoning ability about the norms' effects on their performance.

*Formulating Norms Detection Based on Emotion*. The literature assumed that detection techniques exploit an agent's emotion to trigger its belief on conforming with the majority. Thus, if it does not conform, it feels guilty and detects the norms. However, emotion is represented based on imitation although emotion can be based on context too. Emotions that are based on imitation only trigger beliefs to adapt the majority norms, while when they are based on context, they trigger agents' beliefs to adapt the norms of minority or majority of agents.

*Formulating Norms Assimilation*. The work on norms assimilation is almost nonexistent in normative multiagent systems although it has been theoretically discussed by social science researchers. The goal of norms assimilation can be achieved based on the social theory of assimilation that have been deliberated in the literature, in which the decision to assimilate is influenced by two main elements which are the cost of assimilation and the ability of agents.

*Formulating Norms Removal*. Norms removal is considered as one of the main processes of normative multiagent systems. It describes the situation of a norm's disappearance due to cessation of practice. From our review of the literature, we have not found any formal work in this area.

## 7. Conclusion

This paper reviews the study of normative multiagent systems from two disciplines, which are software agent technology and social norms. Our review shows that agent systems have been established as one of the technologies of this millennium and that multiagent systems are built with several disciplines such as information technology, economics, logic, ecology, biology, philosophy, and sociology.

The review on social norms reveals two categories of norms which are conventional norms and essential norms. In essential norms, there are obligation, prohibition, and permission norms. Upon deliberating these types (obligation, prohibition, and permission norms), we discover and propose a new norm type that we term as recommendation norms. We discover that they are influenced by reward only, while the other types are influenced by reward and penalty.

In norm life cycle, the literature shows that there are three proposed life cycles. However, these researchers emphasized on four main components which are creation, emergence, enforcement, internalization, and learning. 

In normative multiagent systems, we introduce the concept, definition, usage, and the origin of normative multiagent systems. Normative multiagent systems have been studied traditionally by researchers of moral and legal philosophy. The literature reveals ample research that have been conducted on norms architecture such as BOID, BIO, KGP, and OP-RND.

In issues related to normative systems, we discuss norms representation and norms enforcement formats in agent-based system. In norms representation, we notice that there are several kinds of representation such as deontic logic, rule-based systems, binary strings, game theory, decision trees, and temporal logic. In norms enforcement, the norms can be enforced by applying sanctions on violation. This sanction is applied by a third-party enforcement. However, the norms could also be triggered by emotions of shame or guilt in an agent even when the third-party enforcement is absent.

We also review and present the various empirical studies on normative systems and their simulation mechanisms and identify the limitations of these studies. We then review and discuss the limitations of the available models in the literature on norm's life cycle. We exploit those models in developing our own model of norm's life cycle.
